# Polyphenol oxidase silencing avoids protein cross‐linking and enzymatic browning in *Nicotiana benthamiana* leaf extracts

**DOI:** 10.1111/pbi.70202

**Published:** 2025-06-18

**Authors:** Chidambareswaren Mahadevan, Emma C. Watts, Kaijie Zheng, Shi‐jian Song, Renier A. L. van der Hoorn

**Affiliations:** ^1^ The Plant Chemetics Laboratory, Department of Biology University of Oxford Oxford UK

**Keywords:** polyphenol oxidase, browning, phenolics, agroinfiltration, *Nicotiana benthamiana*

Browning of extracts during purification of recombinant proteins from agroinfiltrated leaves is a widely observed phenomenon that is commonly quelled with reducing agents and absorbing materials. In fruits and vegetables, browning results from the oxidation of phenolics into brown quinones which react with themselves and other molecules to form a brown melanin‐like polymer (Figure 1a, Sui *et al*., 2023). The oxidation of phenolics is catalysed by polyphenol oxidase (PPO), which is localized to chloroplasts but oxidizes vacuolar phenolics upon cell disruption. Enzymatic browning in sliced apple and bruised potato has been suppressed by silencing PPO (Carter, 2012; González *et al*., 2020).

**Figure 1:**
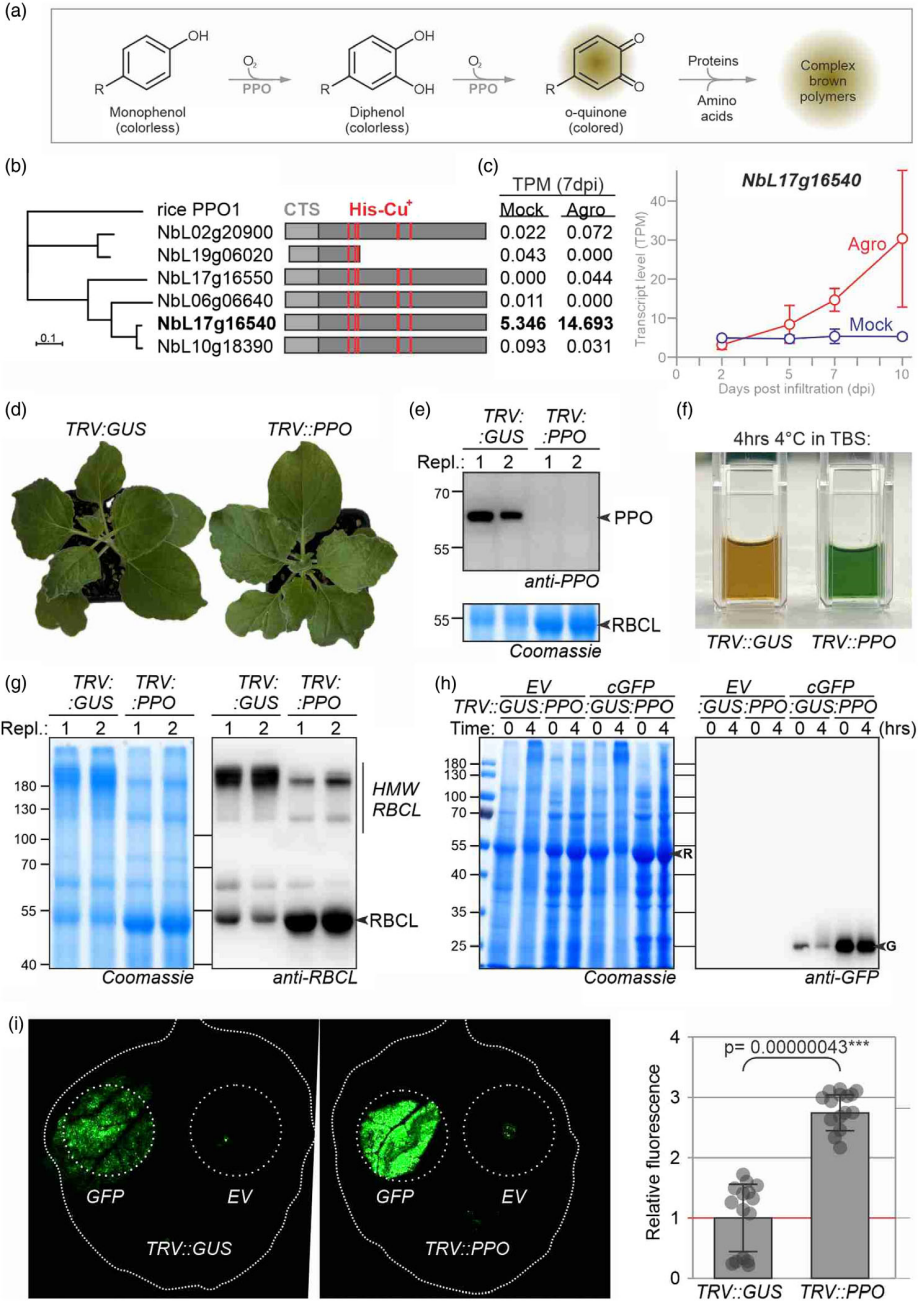
*PPO* silencing avoids browning and cross‐linking and may increase transient expression. (a) PPO oxidizes phenols into brown quinones, which react with themselves and with proteins, resulting in a brown polymer. (b) Neighbor‐joining phylogeny of six *N. benthamiana* PPOs, including rice PPO1 as the out‐group. Domain structures indicate chloroplast targeting signals (CTS) and catalytic His residues (red) that coordinate two copper ions that suspend a molecular oxygen. (c) *PPO* transcript levels in transcripts per million reads (TPM) of mock‐ and agro‐infiltrated leaves (from Grosse‐Holz *et al*., 2018). Error bars represent SE of n = 3 replicates. (d) *PPO* silencing does not affect growth or development. Two‐week‐old plants were agro‐inoculated with tobacco rattle virus (TRV) carrying a 300 bp fragment of GUS or PPO and images were taken 3 weeks later. (e) PPO protein is depleted in *TRV::PPO* plants. Shown are two replicates. (f) Total leaf extracts of *TRV::PPO* plants do not turn brown when incubated for 4 h at 4 °C in Tris‐buffered saline (TBS). (g) Cross‐linking is reduced in leaf extracts of *TRV::PPO* plants when incubated for 4 h at 4 °C. (e–g) Total extracts of two replicates of 5‐week‐old *TRV::GUS* and *TRV::PPO* plants were incubated for 4 h at 4 °C, imaged in a cuvette (f), separated on a protein gel and stained with Coomassie and analysed on Western blot with anti‐PPO antibody (e) or anti‐RBCL antibody (g). R, RBCL; G, GFP. (h) More GFP accumulates upon transient GFP expression in *TRV::PPO* plants. *TRV::PPO* and *TRV::GUS* plants were agroinfiltrated with pEAQ‐HT‐GFP‐P19 and extracts were generated in TBS at 5dpi and incubated for 4 h at 4 °C and analysed by Coomassie staining and Western blot with anti‐GFP antibody. (i) Increased GFP fluorescence in *TRV::PPO* plants. *TRV::PPO* and *TRV::GUS* plants were agroinfiltrated with 35S::GFP and imaged at 5dpi and fluorescence was quantified. Error bars represent SE of *n* = 3 replicates. The *P*‐value was determined with the Student's *t*‐test.

To prevent browning of extracts from agroinfiltrated leaves, we depleted *PPO* transcripts by virus‐induced gene silencing (VIGS). The *N. benthamiana* genome (Ranawaka *et al*., 2023) contains six *PPO* genes, of which five are predicted to encode putative functional enzymes (Figure 1b). However, only *NbL17g16540* is significantly expressed in leaves, and its expression increased upon agroinfiltration (Figure 1c). We cloned a 300 bp fragment to silence *NbL17g16540* and its homeolog (*NbL10g18390*, Figure S1) into RNA2 of the bipartite genome of tobacco rattle virus (TRV). Young *N. benthamiana* plants were co‐agroinoculated with TRV1 and TRV2 carrying fragments of *PPO* or *GUS* (β‐glucuronidase fragment, negative control). Three weeks post‐infiltration, *TRV::PPO* plants showed no growth or developmental phenotypes compared to *TRV::GUS* plants (Figure 1d). Western blot analysis of leaf extracts from these plants confirms that PPO was successfully depleted from *TRV::PPO* plants (Figure 1e).

Importantly, cleared leaf extracts of *TRV::PPO* plants in Tris‐buffered saline (TBS) remained green after 4 h of incubation at 4 °C, in contrast to browning observed in extracts of *TRV::GUS* plants (Figure 1f). When these incubated extracts were separated on protein gels, *TRV::PPO* samples revealed much stronger signals at 55 kDa, whereas *TRV::GUS* samples showed high molecular weight (HMW) signals at >180 kDa (Figure 1g). Western blot analysis revealed that much of the large subunit of ribulose bisphosphate carboxylase large chain (RBCL) runs at HMW in the *TRV::GUS* sample, unlike the 55 kDa signal found in *TRV::PPO* samples (Figure 1g). This indicates that PPO catalyses cross‐linking of RBCL, possibly fixing RBCL tetramers in the multimeric RBCL complex (Duff *et al*., 2000). The HMW signal was less prominent at the *t* = 0 time point (Figure 1h), indicating that cross‐linking occurs during incubation.

To examine the impact of PPO depletion on protein expression and accumulation, we transiently expressed cytoplasmic GFP in *TRV::GUS* and *TRV::PPO* plants. Interestingly, extracts from *TRV::PPO* plants contained significantly higher levels of GFP protein than the *TRV::GUS* control plants (Figure 1h). Further, the intensity of the GFP signal is reduced in *TRV::GUS* extracts upon incubation for 4 h at 4 °C but not in *TRV::PPO* plants. However, we did not detect HMW complexes containing GFP in *TRV::GUS* control plants (Figure 1h). Measuring GFP fluorescence directly from leaves revealed a significant, 2.8‐fold higher GFP fluorescence from *TRV::PPO* plants when compared to *TRV::GUS* plants (Figure 1i), consistent with Western blot analysis and supporting the increased GFP accumulation in *TRV::PPO* plants. Although the underlying mechanism behind the increased transient expression is unclear, this feature might be a valuable additional advantage of *PPO* silencing.

Thus, *PPO* silencing avoids enzymatic browning and protein cross‐linking, and this may increase the yield and quality of purified proteins and improve routinely performed experiments such as co‐immunoprecipitation and metabolic experiments. While *PPO* silencing does not affect plant growth or development, it may reduce immunity to pests and pathogens (Zhang and Sun, 2021). Alternative ways to deplete PPO activity include genome editing, the use of chemical or protein‐based inhibitors or physical methods such as those used in the food industry (Sui *et al*., 2023).

## Funding

This project was financially supported by ERC project 101019324 (CH and RH) and BBSRC projects DDT00230 (EW); BB/W013932/1 (SS, KZ and RH) and BB/Y00969X/1 (KZ and RH).

## Author contributions

RH conceived the project; CM performed most experiments with the help of EW, KZ and SS; RH wrote the manuscript with the help of all authors.

## Conflict of interest

None declared.

## Supporting information


**Data S1** Supplemental materials.
**Table S1** Used plasmids.
**Table S2** Oligonucleotides.
**Figure S1** Alignment of VIGS fragment with 6 *PPO* genes of *N. benthamiana*.

## Data Availability

The data that supports the findings of this study are available in the supplementary material of this article.
