# Supplementary material for: Polyphenol oxidase silencing avoids protein cross‐linking and enzymatic browning in Nicotiana benthamiana leaf extracts
Source: Plant Biotechnol J. 2025 Jun 18;24(1):96–8. doi: 10.1111/pbi.70202 (PMC12854902; doi:10.1111/pbi.70202)
Supplement: Supplementary file 1 — Data S1 Supplemental materials. Table S1 Used plasmids. Table S2 Oligonucleotides. Figure S1 Alignment of VIGS fragment with 6 PPO genes of N. benthamiana. [file PBI-24-96-s001.docx]

Supplemental Materials

**Polyphenol oxidase silencing avoids protein crosslinking and browning in *Nicotiana benthamiana* leaf extracts** Chidambareswaren Mahadevan et al.

***Bioinformatic analysis*** – Transcriptomic data was processed using the High Performance Computing (HPC) compute clusters from the University of Oxford Advanced Research Computing facility (Richards, 2015). Raw sequence reads from a previous transcriptomics study (Grosse‐Holz et al., 2018), were downloaded from Sequence Reads Archives (NCBI) using fasterq from the SRA toolkit v3.0.10 of NCBI. Quality trimming was performed with Trimmomatic v.039 (Bolger et al., 2014) and checked with FastQC v0.12.0. (Wingett and Andrews, 2018). Processed reads were then mapped to the LAB 3.6 genome (Ranawaka et al., 2023) using kallisto v0.50.1 (Bray et al., 2016). The phylogenetic tree of PPO genes was constructed with Geneious Prime® 2024.0.7 (https://www.geneious.com). A distance matrix was built with global alignment and free end gaps, employing the Blosum62 cost matrix. The tree was built using the Neighbor-Joining method with the Jukes-Cantor genetic distance model. *Oryza sativa* polyphenol oxidase I (Gene ID: 4337055) from NCBI was included as the outgroup.

***Plant cultivation conditions -*** Wild-type *Nicotiana benthamiana* (LAB) seeds were sown in a 3:1 mix of soil (Sinclair Modular Seed Peat reduced propagation mix) and vermiculite (Sinclair brand Pro Medium) in 7x7 cm square pots. Seeds were initially grown under high humidity, covered with transparent plastic for 5 days. After uncovering, seedlings were cultivated in the greenhouse at 80–120 µmol/m²/s light, with temperatures set to 21°C at night and 22–23°C during the day, under a 16-hour light cycle. Two-week-old plants were then agroinfiltrated with the respective TRV vectors and transferred to a controlled growth chamber set at 100 µmol/m²/s light, 21°C, and 50–60% relative humidity, also with a 16-hour light regime. Plants were watered three times per week, ensuring even moisture in the pots.

***Construction of plasmids -*** All plasmids used in this study are summarized in Supplemental **Table S1**. A 300 bp fragment of the *PPO* gene (Supplemental **Table S2**) was selected using the VIGS tool (Fernandez-Pozo et al., 2015), synthesized by Twist Biosciences, and cloned into the golden-gate compatible vector TRV2gg (Duggan et al., 2016) using a BsaI restriction enzyme reaction to generate expression plasmids. The resulting plasmid was transformed into *Escherichia coli* DH10β for amplification, purified, and subsequently transformed into *Agrobacterium tumefaciens* GV3101-pMP90. Transformants were selected on LB agar plates containing 25 µM rifampicin, 10 µM gentamycin, and 50 µM kanamycin. A single colony was cultured in liquid LB medium supplemented with the same antibiotics.

**Virus-induced Gene Silencing (VIGS)** - *Agrobacterium* strains containing TRV1 or TRV2 (Supplemental **Table S2**) were grown overnight at 28°C in LB medium supplemented with 25 mg/L rifampicin, 10 mg/L gentamycin, and 50 mg/L kanamycin. Cultures were centrifuged at 3,500 x g for 10 minutes at room temperature, and the resulting pellets were resuspended in agroinfiltration buffer (10 mM MES, pH 5.7, 10 mM MgCl₂, 100 µM acetosyringone) to an OD₆₀₀ of 1.0. TRV1 and TRV2 cultures were then mixed in a 1:1 ratio. Two-week-old *Nicotiana benthamiana* plants were agroinfiltrated with this bacterial suspension using a 1 mL needleless syringe. Three to five weeks post-infiltration, plants were assessed for silencing by observing bleached leaves in *TRV::PDS* positive control plants. Successfully silenced plants were subsequently tested by further agroinfiltration.

***Protein extraction and incubation*** - Proteins were extracted from 1 cm leaf discs, which were punched from the same leaves as above and transferred to 1.5 mL Eppendorf tubes. Each tube received two steel balls (2.3 mm diameter, BioSpec) and 150 µL of extraction buffer (50 mM Tris-HCl, pH 7.6, 250 mM NaCl, 1 mM EDTA, 0.002% Tween-20). Protein extraction was performed using a QIAGEN TissueLyser II with the following settings: 30 strokes, 30 seconds per stroke, repeated twice. The samples were then centrifuged at 13,000 x *g* for 10 minutes at 4°C. Proteins were subsequently incubated in a cold room maintained at 4°C for the 4 hr time-course experiment.

***Western blot analysis -*** The total soluble protein supernatant was mixed at a 1:3 ratio with the 4x gel loading buffer (200 mM Tris-HCl, pH 6.8, 400 mM DTT, 8% SDS, 0.4% bromophenol blue, 40% glycerol) and heated at 95°C for 5 minutes. Proteins were separated on a 12% w/v SDS-PAGE gel, transferred onto a polyvinylidene difluoride (PVDF) membrane using the Trans-Blot Turbo system (Bio-Rad, Hercules, CA), and blocked for 1 hour at room temperature in 5% w/v skimmed milk in phosphate-buffered saline (PBS) with 0.01% v/v Tween-20. The blots were then incubated with primary antibodies: anti-PPO (1:2,500, MyBioSource) or anti-RBCL (1:2,500, Agrisera) or anti-GFP-HRP (1:3,000, Invitrogen) and with the secondary antibody, anti-rabbit (1:5,000, Invitrogen), in 5% w/v skimmed milk in PBS with 0.01% v/v Tween-20. Chemiluminescent signals were detected using the SuperSignal™ West Femto Maximum Sensitivity Substrate (Thermo Fisher Scientific, Waltham, MA, USA).

***GFP Expression, Imaging, and Quantification -*** Agrobacterium strain GV3101(pMP90) carrying the pEAQ-HT-GFP vector (encoding GFP and P19 along with an empty vector, EV) was grown overnight at 28°C in LB medium containing 25 µM rifampicin, 10 µM gentamycin, and 50 µM kanamycin. Cultures were centrifuged at 3,500 x g for 10 minutes at 21°C and resuspended in infiltration buffer (10 mM MES, 10 mM MgCl₂, 100 µM acetosyringone, pH 5.7) to an OD₆₀₀ of 0.5. Expanded leaves of virus-induced gene silencing (VIGS) plants were agroinfiltrated using a needleless syringe. At 5 days post-infiltration (dpi), leaves were detached and scanned for GFP fluorescence on an Amersham Typhoon 5 Biomolecular Imager (GE Healthcare Life Sciences, Little Chalfont, UK) using the 488 nm laser with Cy2 settings. The fluorescence quantification was performed using ImageJ software (Schneider et al., 2012). Statistical significance was assessed using Student’s *t*-test.

**Supplemental Table S1** Used plasmids

| **Plasmid** | **Description** | **Reference** |
| --- | --- | --- |
| *TRV::GUS* | Binary silencing construct for GUS | Duggan et al., 2021 |
| *TRV::PDS* | Binary silencing construct for PDS | Liu et al., 2002 |
| pCM02 | Binary silencing construct for PPO | This work |
| pEAQ-HT-P19 | Binary empty vector construct with P19 | Sainsbury et al., 2009 |
| pEAQ-HT-GFP | Binary expression construct for GFP + P19 | Sainsbury et al., 2009 |

**Supplemental Table S2** Oligonucleotides

| 300bp PPO fragment used for silencing |
| --- |
| TGACAAAAACCTTGACGCTGTTGACAGGAGGAATGTCCTCTTGGGTTTAGGAGGGCTGTATGGCGCAGCTAATCTTGTGCCATTAGCTACTGCTGCTCCTATACCACCTCCTGATCTCAAATCTTGTAGCAAAGCCCATATAAATGACAAAGAGGAGGTTTCATACAGTTGTTGCCCCCCTATCCCAAGTTATATGGACAGCGTTCCATATTACAAGTTTCCTTCTATGCCCAAACTCCGTATTCGGCCCGCTGCTCATGCTGTTGATGAGGAGTACATTGCTAAATACCAGTTAGCCAC |

201 300

**VIGS TGACAAAAACCTTGACG---CTG----TTGACAGGAGGAATGTCCTCTTGGGTTTAGGAGGGCTGTATGGCGCA**

NbL10g18390 ----CCAA-CA-----GTGGCGAGCATGACAAAAACCTTGACG---CTG----TTGACAGGAGGAATGTCCTCTTGGGTTTAGGAGGGCTGTATGGCGCA

**NbL17g16540** ----CCAA-CA-----GTAGCGAGACTGACAAAAACCTTGACG---CTG----TTGACAGGAGGAATGTCCTCTTGGGTTTAGGAGGGCTGTATGGCGCA

NbL06g06640 ----GCGAGCATGAGGGAAACCAGGTTGACGCAATTAAAGAAGGAGCTG----TTGACAGAAGGAATGTCCTTTTGGGTTTAGGAGGGCTGTATGGGGCA

NbL17g16550 ----TCTC-CAATGGTGATGTAAACCAAACTGTTGAAACAAATT--CTG----TTGATAGAAGAAATGTGCTTCTAGGTTTAGGAGGTTTGTATGGTGCT

NbL02g20900 TGACCAAACCAATAACACTTCGAAAAATTCTAATTCTTCAAACAATAAGATCATTGATAGAAGAAACATGCTACTTGGATTAGGAGGTATTTATGGTGCT

NbL19g06020 ------------------TTCAGATGATGCTGATTCTTCAAACAAACAGATCATTGATAGAAGAAACATGCTACTTGGATTAGGAGGCATTTATGGTGTT

301 400

**VIGS GCTAATCTTGTG---------CCATTAGCTACTGCTGCTCCTATACCACCTCCTGATCTCAAATCTTGTAGCAAA------GCCCATATAAATGACAAAG**

NbL10g18390 GCTAATCTTGTG---------CCATTAGCTACTGCTGCTCCTATACCACCTCCTGATCTCAAATCTTGTAGCAAA------GCCCATATAAATGACAAAG

**NbL17g16540** GCTAATCTTGCG---------CCATTAGCTACTGCTGCTCCTATACCACCTCCTGATCTCAAATCTTGTAGCAAA------GCCCATATAAATGACCAAG

NbL06g06640 ACTAATCTTGCG---------CCGTTAGCATCTGCTGCCCCCGTACCACCCCCGGATCTAAAAACATGTGGCCCG------GCCACGATAACGGGTGGTC

NbL17g16550 GCTAATGTTGTA---------CCATTGGCTTCAGCCACTCCCATTCCAGCCCCTACT---ACTTCATGTAGCAAGACTGGCGCCACAATTAAACCTGGTG

NbL02g20900 GCTACTATTGTTGGTGGTCATCCCTTTGCCTTCGCCGCTCCTGTGCCCGGACCTGACGTTTCCAAATGTGGCGCT------GCAAATTTGCCACCAGGTG

NbL19g06020 GCTACTCTTGTTGGTGGTCATCCCTTTGCCTTCGCGGCTCCTGTGCCCGGACCTGACGTTTCCAAATGTGGCCCT------GCAGATTTGCCACCAGGTG

401 500

**VIGS AGGAGGTTTCATACAGTTGTTGCCCCCCTATCCCA---AGTTATATGGACAGCGTTCCATATTACAAGTTTCCTTCTATGCCCAAACTCCGTATTCGGCC**

NbL10g18390 AGGAGGTTTCATACAGTTGTTGCCCCCCTATCCCA---AGTTATATGGACAGCGTTCCATATTACAAGTTTCCTTCTATGCCCAAACTCCGTATTCGGCC

**NbL17g16540** AGGAGGTTTCATACAGTTGTTGCCCCCCAATCCCA---AGTGATATGGACAGCGTTCCATATTACAAGTTTCCTTCTATGCCCAAACTCCGTATTCGGCC

NbL06g06640 CAACTGTATCATATTCTTGTTGCCCCCCTACACCA---GATGATATGGACAGCGTTCCATATTACAAGATCCCTCGCATGTCCAAGCTTCGTAAGAGGCC

NbL17g16550 TACCAGTACCATATTCTTGTTGTCCTCCTCCGCTAAAAATTGATCCTAAGGATATTCCCTATTACAAGTTTCCAACAGGGTCGAAGCTCCGTATTCGACC

NbL02g20900 ---CAGCACCAGTCAACTGTTGTCCTCCAACAACGGCGAATATCATTGAC----TTCCAACTTCCA-----CCACCGTCAACTACCCTCCGCACTCGGCC

NbL19g06020 ---CATCACCAGTCAACTGTTGTCCTCCAACAACGGCGAACATCATCGAC----TTCCAACTTCCA-----CCACCTTCAACCACCCTCCGTACACGGCC

501 600

**VIGS CGCTGCTCATGCTGTTGATGAGGAGTACATTGCTAAATACCAGTTAGCCAC**

NbL10g18390 CGCTGCTCATGCTGTTGATGAGGAGTACATTGCTAAATACCAGTTAGCCACTAGTCGAATGAGGGAACTTGAC---AAAGACCCATTTGACCCTCTTGGC

**NbL17g16540** TGCTGCTCATGCTGCTGATGAGGAGTACATTGCTAAATACCAGTTAGCCACTAGTCGAATGAGGGAACTTGAC---AAAGACCCATTTGACCCTCTTGGC

NbL06g06640 CCCTGCCCAAGACGTGACTGAGGAGTATATAGCCAAGTACCAGTTAGCCACTAGTAAAATGAGGGAATTAGAC---AAAGACCCATTTGATCCTCTTGGC

NbL17g16550 AGCTTCTCATGCCGTGGATGAAGAGTACATGGCTAAGTACAACTTAGCCATTACTAAAATGAAGGAGCTCGATGTTACTGATCCAGATGATCCACGTGGA

NbL02g20900 TGCAGCTCATGCCGCCGATAGTGCCTACATAGAGAAATTCAATAGAGCCATTCAGCTCATGAAACAACTT------CCAGAT---GACGATCCACGTAGC

NbL19g06020 AGCAGCTCATTCCGCCGATAGTGCCTATATAGAGAAATTCAACAGAGCTATTCAGCTCATGAAACAACTT------CCAGAC---GATGATCCACGTAGC

**Figure S1** Alignment of VIGS fragment with 6 *PPO* genes of *N. benthamiana*

**Supplemental References**

**Bolger, A. M., Lohse, M., & Usadel, B.** (2014). Trimmomatic: A flexible trimmer for Illumina sequence data. *Bioinformatics* **30**, 2114–2120.

**Bray, N. L., Pimentel, H., Melsted, P., & Pachter, L.** (2016). Near-optimal probabilistic RNA-seq quantification. *Nat. Biotechn.* **34**, 525–527.

**Dodds I, Chen C, Buscaill P, van der Hoorn RAL.** (2023) Depletion of the *Nb*CORE receptor drastically improves agroinfiltration productivity in older *Nicotiana benthamiana* plants. *Plant Biotechnol. J.* **21**, 1103-1105.

**Duggan C, Tumlas Y, Bozkurt TO.** (2021) A golden-gate compatible TRV2 virus induced gene silencing (VIGS) vector. *Zenodo* 10.5281/zenodo.5666891.

**Fernandez-Pozo N, Rosli HG, Martin GM, Mueller LA.** (2015) The SGN VIGS tool: user-friendly software to design virus-induced gene silencing (VIGS) constructs for functional genomics. *Mol. Plant* **8**, 486-488.

**Grosse‐Holz, F., Kelly, S., Blaskowski, S., Kaschani, F., Kaiser, M., & Van Der Hoorn, R. A. L.** (2018). The transcriptome, extracellular proteome and active secretome of agroinfiltrated *Nicotiana benthamiana* uncover a large, diverse protease repertoire. *Plant Biotechn. J.* **16**, 1068–1084.

**Kourelis J, Marchal C, Posbeyikian A, Harant A, Kamoun S.** (2023) NLR immune receptor-nanobody fusions confer plant disease resistance. *Science* **379**, 934-939.

**Liu Y, Schiff M, Dinesh-Kumar SP.** (2002) Virus-induced gene silencing in tomato. *Plant J.* **31**, 777-786.

**Ranawaka, B., An, J., Lorenc, M. T., Jung, H., Sulli, M., Aprea, G., Roden, S., Llaca, V., Hayashi, S., Asadyar, L., LeBlanc, Z., Ahmed, Z., Naim, F., De Campos, S. B., Cooper, T., De Felippes, F. F., Dong, P., Zhong, S., Garcia-Carpintero, V., Orzaez, D., Dudley, K. J., Bombarely, A., Bally, J., Winefield, C., Giuliano, G., Waterhouse, P. M.** (2023). A multi-omic *Nicotiana benthamiana* resource for fundamental research and biotechnology. *Nat. Plants* **9**, 1558–1571.

**Richards, A.** (2015). University of Oxford Advanced Research Computing.

**Sainsbury F, Thuenemann E C, Lomonossoff G P.** (2009) pEAQ: versatile expression vectors for easy and quick transient expression of heterologous proteins in plants. *Plant Biotechnol. J.* **7**, 682-693.

**Schneider C A, Rasband W S, Eliceiri K W.** (2012) NIH Image to ImageJ: 25 years of image analysis. *Nat. Methods* **9**, 671-675.

**Wingett, S. W., & Andrews, S.** (2018). FastQ Screen: A tool for multi-genome mapping and quality control. *F1000Research* **7**, 1338.
